# Indian *Journal of Orthopaedics*: Exploring a new horizon

**DOI:** 10.4103/0019-5413.73649

**Published:** 2011

**Authors:** Anil K. Jain

**Affiliations:** *Editor, Indian Journal of Orthopaedics, and Professor, Orthopaedics, UCMS, Delhi, India*

The year 2010 is coming to an end. It is one year since we reported the progress of *Indian Journal of Orthopaedics,*^1^ hence the time to analyze where we stand and outline the future directions. We are now moving in the right direction with inclusion in the major bibliographic databases [PubMed, Science Citation Expanded and archiving agencies (Portico, Pubmed Central)]. The manuscript submission has shown a 20% increase over the last year [Figure 1]. The submissions are coming from both Indian and overseas authors in good numbers. Our review decision and publication timelines are constantly improving and we hope to shorten the submission–publication time by increasing the number of issues from current four to six in a year.

**Figure 1:**
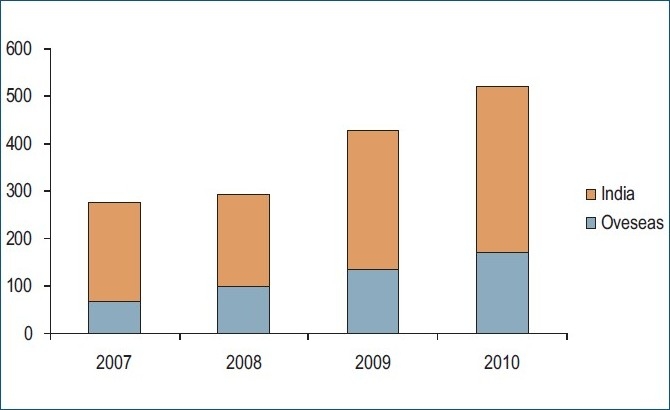
Number of manuscripts submitted to the *Indian Journal of Orthopaedics* during 2007-2010 from India and outside India

This year, we have published 473 pages of scientific contents which include 06 Editorials, 08 Review Articles, 49 Original Articles, 20 Case Reports, 04 Book reviews, 04 Letter to Editors and 03 Obituaries. Three issues (January, April & October) were thematic. All four issues were released on www.ijoonline.com, One month ahead of the scheduled print publication date.

Our website www.ijoonline.com has improved substantially. We have added many new features this year. On release of article/issue on web, the PDF is sent electronically to authors and table of contents to over 15,000 registered users. If any of the articles is cited, a citation alert is sent to the authors. Citations appear on the article page. The website can be accessed by mobile and other handheld devices such as iPad, iPod, etc. On archives of www.ijoonline.com, we can get instant download for table of contents, authors, mapping on Google, number of times each article is downloaded and citation received for particular issue. Most viewed article is marked as popular. One can also browse the entire issue as e-book or flip book.

The journal is included in various other databases and collections such as Rightlinks, Gale CCNGAGE learning, Summons Web Scale Discovery, ALPSP Learned Journal Collection and EBSCO Discovery Service. The number of visitors on our website has increased by over 30% since last year. These visitors are spread globally with over 60% of the visitors coming from USA and Europe. Our citation has increased by about 66% from the last year [Figure 2].

**Figure 2:**
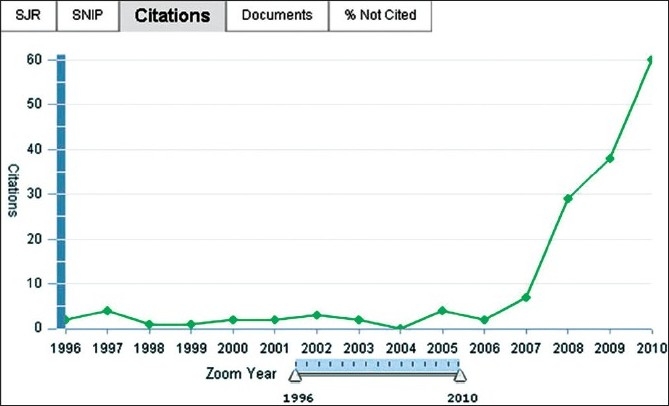
Citation graph shows peak increase in citations of *Indian Journal of Orthopaedics* during 2010 (Source: SCOPUS)

## ARCHIVES (BACK ISSUES)

As promised last year, all back issues available at the Varanasi Office since 1967 have already been digitized and uploaded on www.ijoonline.com and are available on website. Twenty-eight issues which are not available with journal office are to be collected from voluntary donation from the members and are to be digitized and uploaded.

## FACILITIES FOR THE MEMBERS

We have started an e-mail alert to members. When the journal is dispatched, an e-mail, giving the postal details of the issue, is sent so that enquiry can be placed to post and telegraph department on non-receipt of the journal. Every year, we print the list of the journals which are not delivered. A website is created to correct and update addresses online. On any information to journal office, the addresses are updated.

We have a good database of reviewers which is the foundation of peer review process.^2^ This database has a large number of overseas reviewers. We have to work in this area. The manuscripts from our country should definitely be reviewed by reviewers who are working in similar infrastructure and on similar clinical profile of the patients. Hence, there is a need for strong panel of reviewers from India and southeast Asian countries. I appeal you all to contribute with positive intent to the cause of promotion of publication of credible research in India.

## FUTURE

We are increasing the number of issues from four to six per year from the year 2011. Now, *Indian Journal of Orthopaedics* avails submission of articles in large numbers from our learned members and we assure that no article will be delayed/rejected because of scaricity of print pages. This will reduce submission to publication time, and further, our members will have an increased page space to get their submission published. This year, we have introduced “IOA Best Publication award” in Research category beside SN Baxi Best Publication Award in Clinical Research category. For the year 2009, the winner of SN Baxi award was Dr. K.H. Sancheti^3^ from Pune and IOA Best Publication award in Research Category was won by Dr. Ravijot Singh.^4^We intend to start online case report version of *Indian Journal of Orthopaedics* and if the number of case reports are good then we can even print them as supplement. We are likely to get our first impact factor in 2011. To have a high impact factor, we need to ensure that all our future publication will use references published in *Indian Journal of Orthopaedics* for citation. We are planning to conduct workshop on “Research method and scientific writing”. We have conducted one such workshop in Delhi. We can conduct similar workshops in every state. We are bringing out a section of 5–7 pages on Evidence Scan from 2011, wherein one article on “How to evaluate various kinds of published articles” will be printed. The summary of an already published article with commentary will be published. Readers can submit the commentary on a given summary of second published article. The best commentary will be published in the next issue. Next six issues will cover the controversies in skeletal trauma as a special section. Two or three articles covering various controversies in skeletal trauma will be published.

We are thankful to our authors, reviewers, editorial board members, staff at Medknow Publications and office bearers of IOA. We still have to go a long way to explore a new horizon. We believe together we can and together we shall attain new heights.
